# Topics of Public Interest

**Published:** 1904-03

**Authors:** 


### TOPICS OF PUBLIC INTEREST.

### THE PROPOSED OPTOMETRY BILL.

By LUCIEN HOWE, M. D., Buffalo, N. Y.

ON the tenth instant,[1] Senator Wilcox introduced the following bill, which was referred to the Committee on Public Health. It is entitled, An act to amend the Public Health law by defining optometry and regulating the practice thereof.

SECTION 1. Chapter six hundred and sixty-one of the laws of eighteen hundred and ninety-three, entitled "An act in relation to the public health, constituting chapter twenty-five of the general laws," is hereby amended by inserting a new article to be article thirteen thereof and to read as follows:

### ARTICLE XIII.

#### OPTOMETRY.

SEC. 209-a. Definition; application or article.
209-b. State board of examiners.
209-c. Powers of board.
209-d. Examinations; certificates for practitioners.
209-e. Certificates to be recorded and displayed.
209-f. Fees.
209-g. Violations of article.

SEC. 209-a. Definition; application of article.—The practice of optometry is defined to be, the employment of any means, other than the use of drugs, for the measurement of the powers of vision and the adaptation of lenses for the aid thereof. The provisions of this article shall not be construed to apply to physicians duly licensed to practise medicine under the laws of this state, nor to persons who sell spectacles or eyeglasses on prescription from any duly qualified optometrist or physician, nor to dealers in spectacles and eyeglasses who neither practise nor profess to practise optometry.

SEC. 209-b. State board of examiners.—The state board of regents is hereby authorised and directed on or before July 1st, nineteen hundred and four, to appoint a board of examiners in optometry, which shall consist of five persons, who shall have been

1. February 10, 1904.

residents of this state actually engaged in the practice of optometry for at least five years and members of the optical society of the state of New York. For the purpose of such appointment said society shall nominate to said board of regents twice the number of examiners to be appointed, and thereafter similarly for each vacancy or new appointment. The term of each member of said board shall be three years and until his successor is appointed and vacancies shall be filled for unexpired term only; but in the original appointment of the members two shall be appointed for the term of one year, two for two years and one for three years from July 1st, nineteen hundred and four.

Sec. 209-c. Powers of board.—Said board of examiners shall, subject to the approval of the regents, make such rules and regulations not inconsistent with the law, as may be necessary to the proper performance of its duties; and each member thereof may administer oaths or take testimony concerning any matter within the jurisdiction of the board.

Sec. 209-d. Examinations; certificates for practitioners.—Every person desiring to commence or to continue the practice of optometry after January 1st, nineteen hundred and five, except as hereinafter provided, shall take an examination before said board of examiners to determine his qualification therefor. Every candidate successfully passing such examination shall be registered by said board of examiners as possessing the qualifications required by this article and shall receive from said board a certificate thereof; but any person who shall have been continuously engaged in the practice of optometry for more than two years next prior to the passage of this article shall be entitled upon submitting proof thereof to said board of examiners to receive from said board a certificate of exemption from such examination. Every person entitled to a certificate of exemption as herein provided must make application therefor and present the evidence to entitle him thereto, on or before January 1st, nineteen hundred and five, or he shall be deemed to have waived his right to such certificate. Any certificate issued by said board of examiners may be revoked by the board of regents after a hearing and upon due notice to the holder thereof.

Sec. 209-e. Certificate to be recorded and displayed.—Every person to whom a certificate of either registration or exemption shall be issued shall immediately cause the same to be recorded in the clerk's office in the county of his residence and also in the clerk's office of each other county wherein he shall then practise or thereafter commence the practice of optometry; every person practising optometry must also display his certificate of registration or exemption in a conspicuous place in the principal office wherein he practises optometry and whenever required exhibit such certificate to said board of examiners or its authorised representatives.

SEC. 209-f. Fees.—The fee for such examination shall be fifteen dollars; for a certificate of registration ten dollars, and for a certificate of exemption five dollars, to be paid to the board of regents and constitute a fund for expenses made necessary by this article. From the fees so paid, the regents shall cause to be paid all necessary expenses incurred in the administration of this article, including the reasonable compensation of the examiners for their services and their necessary expenses. The fee to be paid to the county clerk for recording a certificate shall be fifty cents.

SEC. 209-g. Violations of article.—Any violations of the provisions of this article shall be a misdemeanor and courts of special session shall have jurisdiction of all such violations. No person not a holder of a certificate of registration or exemption duly issued to him and recorded as above provided, shall, after January 1st, nineteen hundred and five, practice optometry within this state. The practice of or offering to practise optometry or the public representation of being qualified to practise the same by any person not authorised to practise optometry shall be sufficient evidence of a violation of this article.

SEC. 2. Article thirteen of the public health law, renumbered as such article by chapter two hundred and ninety-three of the laws of nineteen hundred and three, is hereby renumbered as article fourteen of said law.

SEC. 3. This act shall take effect immediately.

Senate, No. 378.

In attempting to discuss this bill it is necessary at the outset to appreciate the importance of a fundamental fact which is undisputed and familiar to all practitioners of medicine—namely, that certain imperfections of the vision, even though they can be improved temporarily by glasses are, often in reality, only symptoms of serious diseases of the eye itself, or of the general system. The recognition of these diseases requires some knowledge of anatomy, of physiology, of medicine, and especially of the methods of urinary analysis. If the person to whom such patients apply does not recognise these diseases, and if he contents himself with fitting glasses, he may give to the patient temporary improvement of the vision, but valuable time is lost, which may result in total blindness or in the serious illness, or even in the death of the patient. It is worth while illustrating this fact by one or two examples out of a very considerable number which might be selected.

Glaucoma is well known as one of the most dangerous diseases of the eye. Its earliest symptom is that the patient seeks to increase the strength of his glasses rather more frequently than usual. At this stage the change in the eye itself is so slight that the disease may be easily overlooked. If it be recognised at that early stage and treated promptly and properly, the vision is saved in a very large percentage of cases. On the other hand, the greater the delay the more certain is blindness, absolute and total, which, moreover, is often accompanied by severe pain.

Again, while headaches and pain in the eyes are usually the result of so-called "eye-strain," on the other hand in certain instances these symptoms are not dependent upon the eyes primarily, but are due to derangements of the stomach or other internal organs, or, it may be, to an early stage of certain diseases of the brain.

If the person who prescribes glasses for headaches of this kind does not also recognise with some degree of exactness the existence of the disease in these other organs, or at least does not know the other symptoms characterising these other diseases, the patient is not only subjected to unnecessary annoyance and expense in obtaining glasses, but time is lost during which the patient might be receiving proper treatment for that portion of the body which is primarily at fault.

Or again, in the formation of cataract the vision decreases, and changes in the glasses are necessary. But certain forms of cataract are directly the result of diabetes. If the examiner is not sufficiently familiar with the symptoms of this disease and capable of making a urinary analysis, the diabetes is overlooked, its treatment by a physician is neglected, and thus life is endangered. Or still again, failure of vision is not an infrequent symptom of certain forms of Bright's disease. If the examiner is not able to make a chemical examination of the urine and thus recognise the real cause of the difficulty, very valuable time is lost in attempts to improve the vision, and the life of the patient is placed immediately and seriously in danger.

Other examples could be given to show how imperfect vision is only a symptom of more important changes which take place in the eye itself, or in some other organ of the body.

In view of this there seems to be no question but that any one who attempts to prescribe glasses should also have a knowledge of anatomy—at least of the eye,—of physiology, enough to recognise the relation between the eye and other parts of the body, that he should have a considerable knowledge of medicine and certainly a thorough knowledge of urinary analysis. *In a word, the prescribing of glasses is really a branch of the practice of medicine.*

The objections to the bill advocated by the Optical Society are:

First, that it does not sufficiently define what optometry is. It does not state what knowledge, if any, is required of the instruments to be used, other than to recognise what they are. It does not even require a regents certificate or any knowledge of optics.

Second, even if a thorough knowledge of optics were demanded, that would be entirely insufficient for the responsibility of prescribing glasses, unless a considerable degree of medical knowledge was added.

Third, the law already requires dentists and pharmacists, and, above all, physicians, to pass examinations, determined by competent judges, of the work required in their respective departments, and it is only right that those who propose to practise medicine under the name of optometry should also be obliged to pass medical examinations whose standard is determined not by special legislation, but in accordance with the system of professional studies already adopted in this state.

Fourth, it is unjust to physicians thus to lower the standard of medical education, and they have already protested against it.

Fifth, even if the bill passes, it does not give to opticians any greater privileges than they now enjoy, but on the other hand it does allow all sorts of frauds and fakirs to continue their practice just as at present, provided only they have done so for two years.

When preparing this bill the opticians sent to physicians a circular letter, the wording of which was such as to indicate that the bill was an effort to raise the standard of practice in optometry. They enclosed a postal card asking physicians who favored the bill to return the card with an expression of approval. A considerable number of physicians signed the card, but on examining the bill and the accompanying letter more closely, it was found by many that they had signed the card under a misapprehension of the facts, and they are now ready to express their condemnation of the bill. In view of the above facts it seems better for the health of the public, and more fair and just to all practising physicians to have the proposed optometry bill defeated by an overwhelming vote.

Another aspect of this question, however, deserves consideration. The Optical Society contains about 200 members, many of whom are conscientious and ambitious to see a real elevation in the standard of their calling. The question therefore arises naturally, is it not possible to accomplish this, and can there not be some coöperation between the medical profession and opticians which will be for the credit of both and also for the benefit of the public? Apparently such a solution of the question is possible. In order to accomplish this two things are necessary. These are:

First, the establishment of optical schools or colleges in connection with medical colleges already established. Some of these medical colleges have already departments of pharmacy or dentistry. It would not be difficult to add also a school of optics or "optometry." The course of study should cover at least three years. It could begin with the usual regents' examinations. The students could study the anatomy of the eye, its physiology, its relation to the general system, some physiology, some medicine, and urinary analysis. The course in optics could be complete and thorough, and they could have practical experience in the grinding of lenses, making and fitting frames and all that pertains to that part of the art.

The fact that there are already in existence many small schools in this country which give short courses in optics indicates that there would be a sufficient number of students ready to qualify themselves more thoroughly.

The higher class of opticians, being anxious to raise the standard in that department of education, would naturally be among the founders of such schools and would become valuable co-workers as instructors.

On the completion of such a course the student would receive a diploma with the seal of the college or university, stating his proficiency as a graduate optician.

Second, a law could be passed establishing a board of examiners in optics or in "optometry." This board could be formed in the same manner as the State Board of Medical Examiners, and have the same functions and jurisdictions. The law, if properly worded, could also be within the limits of the state constitution in requiring that after a given date no one would be allowed to prescribe glasses who had not received a license to do so either from the State Board of Medical Examiners or from the board of examiners in optics. An optician thus qualified by three years' study could be trusted to prescribe glasses according to his discretion, though not to use drugs or make operations.

In any such law it would be safe and wise to allow a jeweler or anyone to prescribe glasses for a person when the vision of neither eye is less than a fourth or a third of the normal, as that always has been done and probably would be done in spite of any law that could be framed.

The practical operation of such a plan would be to create three classes of persons who have to do with the prescribing of glasses and the treating of diseases of the eyes, namely:

First, the physician or surgeon or especially the ophthalmic surgeon, who would have studied medicine for the usual four years, and, in addition, qualified himself by clinical study of the diseases of the eye. He would be familiar with drugs and instruments and be expected to use them with special skill.

Second, the graduate or registered optician, who had studied the subject for three years, who is permitted by law to prescribe glasses, no matter how imperfect the vision of the patient may be, but who does not practise surgery, and uses no drugs.

Third, the simple dealer in glasses, the jeweler, in fact anyone who wishes to give or sell glasses to persons when the vision of neither eye is less than a certain amount, say one-half of normal.

Such a plan would be in harmony with that followed for instruction in pharmacy and dentistry, it would lift the standard of education among opticians at once to a high level, it would not interfere materially with the small optical dealer or jeweler optician, and in every way it would be an advantage to the public. In view of the rapid advances in this department of ophthalmology it is probable that the work of the optician will be recognised by law as a special branch sooner or later, as is the work of the dentist or pharmacist, and when this is done, the law which is then enacted should be vastly better than the one now proposed by the Optical Society.

183 Delaware Avenue.

---

### EXAMINATIONS FOR ARMY SERVICE.

The examination of applicants for appointment as assistant surgeon in the United States Army will be resumed in Washington immediately after the close of the present session of the Army Medical School; it will embrace the full examination (as heretofore), at the conclusion of which those found qualified will be commissioned. The examining board will probably reassemble about the middle of April next, and those desiring to present themselves before the board should make application at once. Applicants are restricted in age to thirty years, and one year's hospital experience or its equivalent in private practice is required. Full information as to the requisite qualifications for appearance for examination, method of application, nature and scope of examination, and the like, may be obtained upon application to the Surgeon General, U. S. Army, Washington, D. C.

---

### EXAMINATION FOR THE PUBLIC HEALTH AND MARINE HOSPITAL SERVICE.

A board of officers will be convened to meet at the Bureau of Public Health and Marine Hospital Service, 3 B street, S. E., Washington, D. C., Monday, April 4, 1904, at 10 o'clock a. m. for the purpose of examining candidates for admission to the grade of assistant surgeon in the Public Health and Marine Hospital Service. Candidates must be between twenty-two and thirty years of age, graduates of a reputable medical college, and must furnish testimonials from responsible persons as to their professional and moral character. The following is the usual order of the examinations: First, physical; second, oral; third, written; fourth, clinical. In addition to the physical examination, candidates are required to certify that they believe themselves free from any ailment which would disqualify them for service in any climate.

The examinations are chiefly in writing, and begin with a short autobiography of the candidate. The remainder of the written exercises consists in examination on the various branches of medicine, surgery and hygiene. The oral examination includes subjects of preliminary education, history, literature, and natural sciences. The clinical examination is conducted at a hospital, and when practicable, candidates are required to perform surgical operations on a cadaver. Successful candidates will be numbered according to their attainments on examination, and will be commissioned in the same order as vacancies occur.

Upon appointment the young officers are, as a rule, first assigned to duty at one of the large marine hospitals, as at Boston, New York, New Orleans, Chicago, or San Francisco. After five years' service, assistant surgeons are entitled to examination for promotion to the grade of passed assistant surgeon. Promotion to the grade of surgeon is made according to seniority, and after due examination as vacancies occur in that grade.

Assistant surgeons receive sixteen hundred dollars; passed assistant surgeons, two thousand dollars; and surgeons, twenty-five hundred dollars a year. When quarters are not provided, commutation at the rate of thirty, forty and fifty dollars a month, according to grade, is allowed. All grades above that of assistant surgeon receive longevity pay, ten per centum in addition to the regular salary for every five years' service up to forty per centum after twenty years' service. The tenure of office is permanent. Officers traveling under orders are allowed actual expenses.

For further information, or for invitation to appear, before the board of examiners, address.

SURGEON-GENERAL,
*Public Health and Marine Hospital Service, Washington, D. C.*

February 16, 1904.

